# Glomerular Injury Is Associated with Severe Courses of Orthohantavirus Infection

**DOI:** 10.3390/pathogens13080693

**Published:** 2024-08-16

**Authors:** Christian Nusshag, Josephine Uhrig, Gefion Gruber, Pamela Schreiber, Martin Zeier, Ellen Krautkrämer

**Affiliations:** Department of Nephrology, University of Heidelberg, 69120 Heidelberg, Germany

**Keywords:** hantavirus, acute kidney injury, AKI, proteinuria, hemorrhagic fever with renal syndrome, HFRS, glomerular injury, podocytes

## Abstract

Hemorrhagic fever with renal syndrome (HFRS) induced by Eurasian pathogenic orthohantaviruses is characterized by acute kidney injury (AKI) with often massive proteinuria. The mechanisms of the organ-specific manifestation are not completely understood. To analyze the role of glomerular and tubular damage in kidney injury induced by HFRS, we measured specific markers in urine samples of patients with acute Puumala virus (PUUV) infection and determined their correlation with disease severity. Levels of α1-microglobulin (α1-MG) and kidney injury molecule 1 (KIM-1), which is expressed by injured tubular epithelial cells, were measured to detect tubular dysfunction and injury. Immunoglobulin G (IgG) and the podocyte specific protein nephrin served as markers for glomerular injury. All four markers were elevated on admission. Markers of glomerular injury, IgG and nephrin, correlated with markers of disease severity such as length of hospitalization, serum creatinine, and proteinuria. In contrast, tubular injury did not correlate with these severity markers. Our results demonstrate that hantavirus infection induces both glomerular and tubular injury early in the clinical course. However, the glomerular dysfunction and podocyte injury seem to contribute directly to disease severity and to play a more central role in HFRS pathogenicity than direct damage to tubular epithelial cells.

## 1. Introduction

Infections with Eurasian hantaviruses of the genus Orthohantavirus cause hemorrhagic fever with renal syndrome (HFRS), which is characterized by acute kidney injury (AKI) with often massive proteinuria [1]. Infections with hantaviruses exhibit a broad range of severity and vary between different hantaviruses, but also patient-specific differences are observed. The involvement of the kidney is a hallmark in apparent infections. However, the underlying mechanisms of the organ-specific injury in HFRS are not well understood.

Light microscopy analysis of kidney biopsies of patients with HFRS reveals a tubulointerstitial nephritis. However, electron microscopy studies demonstrate morphological changes in the tubular apparatus and the glomeruli [2,3,4]. In addition, immunofluorescence studies show disruption of cell-to-cell contacts of cells within the glomerular filtration barrier and the tubular epithelium [5]. Different cell types of the kidney are target cells of HFRS-causing hantaviruses and infection results in cell type-specific functional consequences [5,6,7,8,9]. Several studies characterizing AKI and proteinuria indicate an involvement of the tubular epithelium and the glomerular apparatus in HFRS [4,10,11,12,13]. A plethora of plasma, serum, and urinary markers has been described to be elevated in HFRS and to be associated with disease severity [14,15,16,17,18,19,20]. Standard markers of kidney function and also more and more novel markers have been identified and analyzed in patients suffering from AKI induced by hantaviral infection and indicate a glomerular and tubular injury [4,16,21,22,23,24,25].

To analyze the underlying mechanisms of hantavirus-induced AKI and proteinuria in patients infected with Puumala virus in more detail, we analyzed urinary markers for glomerular and tubular injury in parallel. We used the standard markers α1-MG and IgG for tubular and glomerular dysfunction, respectively. In addition, we analyzed two cell type-specific markers to identify direct cellular damage during HFRS: nephrin as marker of podocyte damage and KIM-1 for the detection of injury to tubular epithelial cells. The analysis of the correlation between these four markers and disease severity will evaluate the role of glomerular and tubular dysfunction and will detect cell type-specific direct injury to podocytes and tubular epithelial cells.

## 2. Materials and Methods

### 2.1. Patients

Patients (*n* = 22) with serologically confirmed acute infection with Puumala virus (PUUV) and hospitalized in the Department of Nephrology, University of Heidelberg, Germany were included. All patients met the case definition of acute hantavirus infection of the German Robert Koch Institute. Clinical data were analyzed through a review of medical charts of the Department of Nephrology. An age- and gender-matched healthy control group (*n* = 10) was recruited. Informed written consent was obtained from all participants and the study was approved by the Ethics Committee of the University Hospital of Heidelberg, Germany, and it adhered to the Declaration of Helsinki. As there are currently no targeted antiviral treatment options, all patients were treated symptomatically with antipyretics and pain relievers (no non-steroidal anti-inflammatory drug (NSAID)).

### 2.2. Enzyme-Linked Immunosorbent Assay (ELISA)

Levels of KIM-1 and nephrin in urine samples were quantified by human urinary KIM-1 Quantikine ELISA Kit (R&D Systems, Minneapolis, MN, USA) and human nephrin ELISA kit (Elabscience Biotech Co., Ltd., Houston, TX, USA), respectively. Assays were performed according to the manufacturer’s instructions. Levels of IgG and α1-MG in urine samples were measured in the accredited Central Laboratory of the University Hospital, Heidelberg.

### 2.3. Statistical Analysis

Data were analyzed using GraphPad Prism 5.0 (GraphPad Software, Boston, MA, USA). Normal distribution was tested with the Shapiro–Wilk test. Comparisons between groups were performed using Mann–Whitney or Student’s *t*-tests. Correlation was assessed by calculating Spearman’s correlation coefficients. *p* values of <0.05 were considered significant. * *p* < 0.05, ** *p* < 0.01, *** *p* < 0.001, **** *p* < 0.0001.

## 3. Results

### 3.1. Levels of Glomerular and Tubular Injury Markers in Patients with HFRS

**Figure 1:**
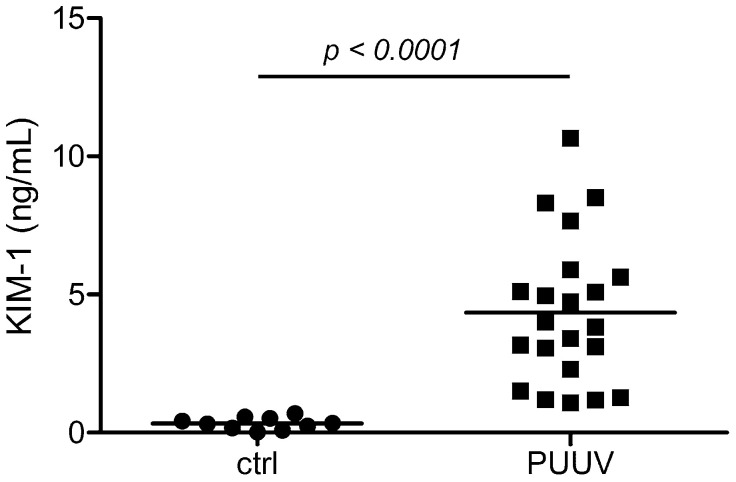
Urinary KIM-1 levels in patients with PUUV-HFRS (*n* = 22) on admission and a healthy age- and gender-matched control group (*n* = 10). Horizontal lines indicate means.

We analyzed levels of KIM-1 in urinary samples of patients with acute PUUV infection. The patients exhibited the typical laboratory characteristics of HFRS: levels of serum creatinine, lactate dehydrogenase (LDH), C-reactive protein (CRP), and the number of leukocytes were elevated. The number of thrombocytes and levels of serum albumin were decreased (Table 1). As observed in our previous study, levels of α1-MG, IgG and nephrin were elevated on admission [4]. To analyze if a direct tubular damage was present on admission, we analyzed levels of KIM-1, a marker of injured proximal tubular epithelial cells, in parallel. Levels of urinary KIM-1 were elevated in patients with acute hantavirus infections compared to an age- and gender-matched control group (mean ± SD: 4.346 ng/mL ± 2.652 ng/mL versus mean ± SD: 0.337 ng/mL ± 0.215 ng/mL; *p* < 0.0001) (Figure 1). No difference in urinary KIM-1 levels between men and females existed (mean ± SD: 4.683 ng/mL ± 2.685 ng/mL versus mean ± SD: 2.830 ng/mL ± 2.154 ng/mL; *p*: 0.2142).

### 3.2. Course of Levels of KIM-1 in HFRS

**Figure 2:**
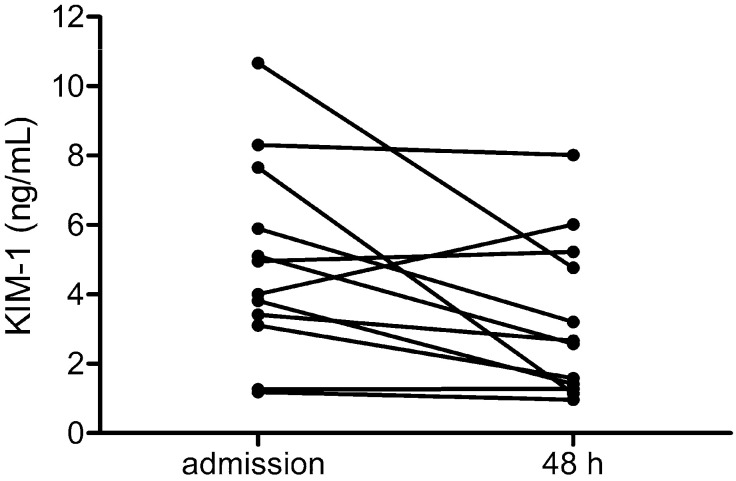
Levels of urinary KIM-1 were measured in patients with PUUV-HFRS (*n* = 12) on admission and 48 h after admission.

To analyze the course of tubular damage, we measured KIM-1 levels on admission and two days later (Figure 2). Levels were highest early in the clinical course and were mostly decreasing after admission as revealed by measuring urinary KIM-1 levels on admission and 48 h post admission in 12 patients.

**Figure 3:**
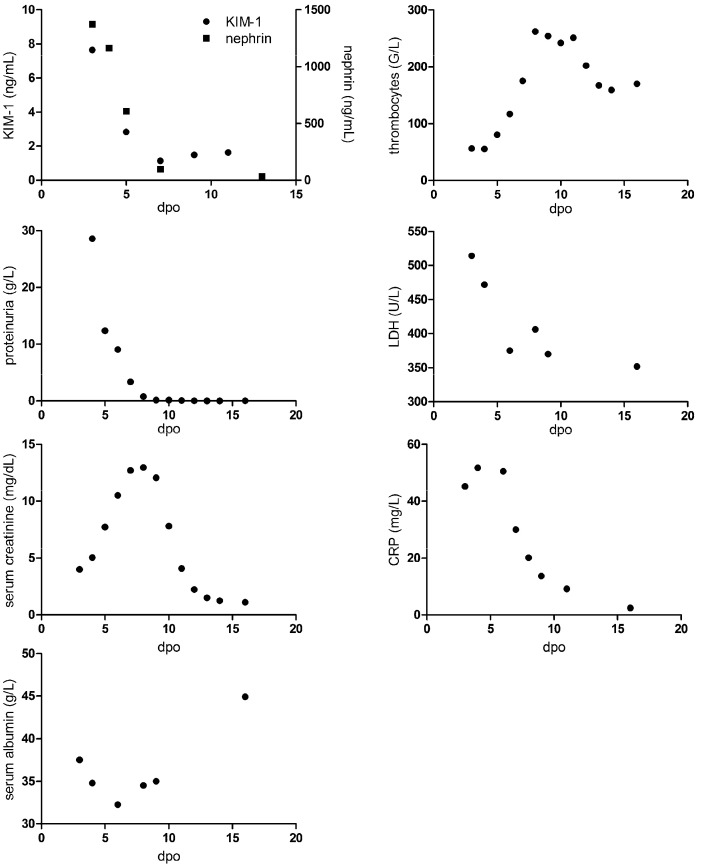
Course of urinary KIM-1 and nephrin levels and laboratory parameters in a patient with PUUV-HFRS. dpo: days post onset of symptoms.

We compared the course of KIM-1 levels with other laboratory parameters during hospitalization (Figure 3). The course of KIM-1 levels was paralleled to the course of nephrin, proteinuria, and LDH. These levels were highest on admission and were decreasing afterwards. In addition, number of thrombocytes were lowest on admission. In contrast, peak levels of serum creatinine and CRP as well as nadir of serum albumin were observed later in the clinical course. Together, we observed a similar kinetic profile of podocyte and tubular injury together with proteinuria.

### 3.3. Correlation of Tubular and Glomerular Injury with Disease Severity

In a next step, we analyzed, if KIM-1 levels in urine samples differ between patients with moderate and severe course. We classified two cohorts according to the median duration of hospitalization in patients with moderate course (LOS ≤ 5 days) and severe course (LOS > 5 days) (Table 1). As described previously, gender ratio between the cohorts did not differ [26].

No differences in age, maximum leukocytes, CRP, and LDH levels or days from onset to admission were observed between the group with moderate and severe course. In contrast, levels of maximum serum creatinine and proteinuria were higher in the group with severe course, whereas minimum levels of serum albumin and thrombocytes were lower in this group. We also analyzed the levels of glomerular and tubular markers in patients with moderate and severe disease. Interestingly, glomerular markers (IgG and nephrin) differed between the two groups whereas no differences were observed for the tubular markers α1-MG and KIM-1.

Glomerular damage was more pronounced in the group with severe disease while tubular markers exhibited no differences. KIM-1 and nephrin are markers of direct tubular epithelial and podocyte injury, respectively. Cellular damage to glomerular or tubular cells may contribute to the clinical picture of HFRS. To examine the relationship between tubular and glomerular injury and disease severity, we analyzed the correlation of tubular injury marker KIM-1 with laboratory parameters and compared the correlation with the results for nephrin (Table 2). As observed in our previous studies, nephrin levels correlated with LOS, maximum levels of serum creatinine, proteinuria, CRP, and minimum levels of thrombocytes and serum albumin [4,26]. In contrast, KIM-1 levels did not correlate with any of the analyzed parameters indicating that tubular injury is not directly associated with disease severity. Interestingly, levels of KIM-1 and nephrin also did not correlate. We conclude from these findings that both glomerular and tubular structures are injured early after onset of symptoms, but the extent of tubular and glomerular injury does not correlate.

Nephrin and KIM-1 represent direct markers of cellular damage in podocytes and tubular epithelium, respectively. In contrast, IgG indicates a loss of function in glomerular filtration and α1-MG in tubular reabsorption capacity. Therefore, we compared the correlation of the standard markers α1-MG for tubular and IgG for glomerular dysfunction with laboratory parameters (Table 3).

α1-MG correlated with maximum levels of CRP and leukocytes as well as with minimum levels of platelets. In contrast, IgG levels showed correlation with LOS, maximum proteinuria, and maximum serum creatinine levels. IgG demonstrated a correlation pattern that is similar to nephrin. In addition, α1-MG and IgG correlated with nephrin, but not with KIM-1.

Together, our results demonstrate tubular as well as glomerular injury with a similar kinetic profile in the clinical course, but only the glomerular injury and dysfunction showed a strong association with disease severity.

## 4. Discussion

Infections with pathogenic hantaviruses are characterized by specific organ manifestation and a broad range of disease severity. Our results demonstrate injury to tubular epithelial cells, which occurs in parallel to the glomerular injury. Both glomerular and tubular markers of injury increase early after onset and decrease afterwards, because Puumala virus disease is a self-limiting infection. The severity of the acute infection shows a strong correlation with direct glomerular injury [4,26].

To understand the underlying mechanisms, it is necessary to examine the AKI in more detail by analyzing the local effects in the kidney using cell type-specific markers. In contrast to α1-MG and IgG, which indicate a loss of function in glomeruli or tubules, detection of cell type-specific markers such as nephrin and KIM-1 in urine samples of patients with acute HFRS may help to identify direct cellular injury and processes that are central in hantavirus infection. The direct podocyte injury identified by elevated levels of urinary nephrin is strongly associated with disease severity. Simultaneously to the elevation of nephrin, we observed an increase of KIM-1 levels indicating a tubular damage that is paralleled to podocyte injury. However, we could not demonstrate an association between tubular injury and disease severity. Contrasting results were observed for neutrophil gelatinase-associated lipocalin (NGAL) levels in HFRS [22]. NGAL is used as a marker of injury to the distal convoluted tubule [27]. For urinary NGAL, a correlation with plasma creatinine and hospital stay was observed [22] leading to the author’s assumption that Puumala virus infection mainly causes tubulointerstitial injury. A possible explanation for the correlation of NGAL but not KIM-1, with disease severity may be the origin of the proteins. In contrast to KIM-1, which is predominantly expressed by tubular epithelial cells, NGAL expression is induced in neutrophils by cytokines in inflammatory processes [28]. Neutrophilia and neutrophil activation are observed in HFRS and may contribute to elevated urinary NGAL [26,29,30,31]. Therefore, it is necessary to analyze more markers in parallel and to elucidate the origin of elevated proteins. The damage to glomerular and tubular cells may be induced by direct effects of infection and/or the activation of the immune system. The infection of cells with hantaviruses influences their transcription and expression profile [32,33]. For renal cell types the up regulation of kidney injury proteins has been demonstrated. Comparison of the proteome profile of urinary samples of HFRS patients with the proteome profile of infected mesangial cells revealed a partial overlap in proteins, which are deregulated in hantavirus infection in vivo and in vitro [8]. Cabrera et al. observed elevated levels of heparinase (HPSE), a marker of AKI, in urine samples of HFRS patients and in supernatants derived from in vitro infected podocytes [25]. These findings indicate that the direct infection of kidney cells may trigger glomerular injury.

In addition, the presence of viral RNA in urine is associated with severe AKI in HFRS [34]. To what extent direct infection and viral replication in glomerular and tubular cell types contributes to proteinuria in hantavirus infection remains to be elucidated. In vitro infection studies as well as detailed analysis of urinary samples will help to identify target cells and the underlying local signaling mechanisms leading to cellular dysfunction.

## Figures and Tables

**Table 1:** Characteristics and laboratory parameters of patients with acute hantavirus infection (*n* = 22) grouped according to the length of hospital stay (LOS) in moderate (LOS ≤ 5 days) or severe disease (LOS > 5 days).

	All Patients (*n* = 22) Median (Range)	Moderate (*n* = 14) Median (Range)	Severe (*n* = 8) Median (Range)	*p* Value
Gender (male/female)	18/4	11/3	7/1	1.000
Age (years)	34 (22–61)	36.5 (22–61)	33.5 (24–44)	0.3524
LOS (days)	5 (3–15)	4.5 (3–5)	8 (6–15)	0.0001 ***
SCre_max_ (mg/dL)	5.41 (1.93–18.02)	4.820 (1.930–9.630)	9.935 (2.270–18.02)	0.0027 **
SAlb_min_ (g/L)	35.10 (22.7–42.30)	36.10 (33.40–42.30)	33.3 (22.7–36.8)	0.0098 **
Platelets_min_ (10^9^/L)	110.5 (21–291)	135 (55–291)	75.5 (21–154)	0.0220 *
Leukocytes_max_ (10^9^/L)	10.33 (4.92–14.54)	10.61 (5.36–14.54)	9.28 (4.92–11.67)	0.2295
Proteinuria_max_ (g/L)	3.519 (0.109–16.93)	1.286 (0.109–9.831)	4.217 (0.2490–16.93)	0.0474 *
LDH_max_ (U/L)	385 (262–514)	372 (262–500)	414 (269–514)	0.7662
CRP_max_ (mg/L)	59.95 (17.2–150.1)	54.65 (17.2–141.3)	68.25 (29.8–150.1)	0.4306
Adm dpo (days)	6 (3–9)	6 (4–9)	6 (3–7)	0.3740
Nephrin_adm_ (ng/mL)	141.6 (10.3–2571)	95.49 (10.3–475.6)	411.9 (172.7–2571)	0.0020 **
IgG_adm_ (mg/L)	455.0 (4.1–3620)	194 (4.1–1540)	643 (268–3620)	0.0140 *
KIM-1_adm_ (ng/mL)	3.91 (1.084–10.66)	3.135 (1.084–10.66)	5.033 (1.193–8.503)	0.1087
α1-MG_adm_ (mg/L)	24.85 (6.2–127)	20.3 (7.2–42)	31.3 (6.2–127)	0.2673

SCre: serum creatinine, SAlb: serum albumin, adm: levels on admission, max: maximum levels, min: minimum levels, dpo: days post onset. Bold values indicate statistical significance. * *p* < 0.05, ** *p* < 0.01, *** *p* < 0.001.

**Table 2:** Correlation analysis of urinary KIM-1 and nephrin levels with clinical parameters in patients with HFRS.

Parameter	KIM-1_adm_ (ng/mL)			Nephrin_adm_ (ng/mL)		
	R	CI	*p* Value	R	CI	*p* Value
Age (years)	−0.3144	−0.6576–0.1367	0.1541	−0.0393	−0.4845–0.4221	0.8695
LOS (days)	0.2014	−0.2533–0.5831	0.3688	0.8715	0.6982–0.9483	<0.0001 ****
SCre_max_ (mg/dL)	0.1846	−0.2694–0.5716	0.4107	0.6947	0.3518–0.8732	0.0007 ***
Proteinuria_max_ (g/L)	0.1790	−0.2749–0.5676	0.4254	0.7616	0.4812–0.9006	<0.0001 ****
LDH_max_ (U/L)	0.0311	−0.4070–0.4575	0.8909	0.0887	−0.3804–0.5216	0.7099
CRP_max_ (mg/L)	0.1248	−0.3253–0.5288	0.5800	0.4466	−0.0091− 0.7487	0.0484 *
Platelets_min_ (10^9^/L)	0.1243	−0.3258–0.5285	0.5816	−0.5515	−0.8041–−0.1303	0.0117 *
Leukocytes_max_ (10^9^/L)	−0.1722	−0.5629–0.2813	0.4434	−0.0165	−0.4669–0.4406	0.9448
SAlb_min_ (g/L)	0.1767	−0.2770–0.5660	0.4314	−0.4556	−0.7536–−0.0023	0.0435 *
Nephrin_adm_ (ng/mL)	0.2496	−0.2303–0.6319	0.2885	−	−	-

Bold values indicate statistical significance. * *p* < 0.05, *** *p* < 0.001, **** *p* < 0.0001.

**Table 3:** Correlation analysis of urinary α1-MG and IgG levels with clinical parameters in patients with HFRS.

Parameter	α1-MG_adm_ (mg/L)			IgG_adm_ (mg/L)		
	R	CI	*p* Value	R	CI	*p* Value
Age (years)	−0.3752	−0.7084–0.0947	0.1030	−0.2319	−0.6204–0.2481	0.3253
LOS (days)	0.3147	−0.1623–0.6725	0.1765	0.7572	0.4621–0.9013	0.0001 ***
SCre_max_ (mg/dL)	0.3714	−0.0991–0.7062	0.1069	0.6311	0.2483–0.8434	0.0028 **
Proteinuria_max_ (g/L)	0.4226	−0.0387–0.7354	0.0634	0.9026	0.7601–0.9623	<0.0001 ****
LDH_max_ (U/L)	0.2722	−0.2073–0.6462	0.2457	0.2978	−0.1668–0.6542	0.2022
CRP_max_ (mg/L)	0.5684	0.1544–0.8126	0.0089 **	0.1136	−0.3588–0.5396	0.6335
Platelets_min_ (10^9^/L)	−0.5546	−0.8056–−0.1346	0.0112 *	−0.2853	−0.6544–0.1936	0.2228
Leukocytes_max_ (10^9^/L)	0.5865	0.1807–0.8216	0.0066 **	−0.0888	−0.5216–0.3804	0.7098
SAlb_min_ (g/L)	−0.0211	−0.4704–0.4370	0.9298	−0.2181	−0.6114–0.2616	0.3556
Nephrin_adm_ (ng/mL)	0.5519	0.1308–0.8043	0.0116 *	0.7672	0.4808–0.9057	<0.0001 ****
KIM-1_adm_ (ng/mL)	0.3805	−0.0887–0.7115	0.0980	0.2836	−0.1954–0.6533	0.2257
IgG_adm_ (mg/L)	0.4332	−0.0256–0.7413	0.0564	−	−	−

Bold values indicate statistical significance. * *p* < 0.05, ** *p* < 0.01, *** *p* < 0.001, **** *p* < 0.0001.

## Data Availability

The data supporting the findings of this study can be obtained from the corresponding author upon reasonable request.
